# Dynamic Organization of Hierarchical Memories

**DOI:** 10.1371/journal.pone.0162640

**Published:** 2016-09-12

**Authors:** Tomoki Kurikawa, Kunihiko Kaneko

**Affiliations:** 1 Lab for Neural Circuit Theory, RIKEN Brain Science Institute, Wako, Saitama, 351-0198, Japan; 2 Research Center for Complex Systems Biology, University of Tokyo, Meguro, Tokyo, 153-8902, Japan; 3 Research Fellow of Japan society for the Promotion of Science, Chiyoda, Tokyo, 102-0083, Japan; Tokai University, JAPAN

## Abstract

In the brain, external objects are categorized in a hierarchical way. Although it is widely accepted that objects are represented as static attractors in neural state space, this view does not take account interaction between intrinsic neural dynamics and external input, which is essential to understand how neural system responds to inputs. Indeed, structured spontaneous neural activity without external inputs is known to exist, and its relationship with evoked activities is discussed. Then, how categorical representation is embedded into the spontaneous and evoked activities has to be uncovered. To address this question, we studied bifurcation process with increasing input after hierarchically clustered associative memories are learned. We found a “dynamic categorization”; neural activity without input wanders globally over the state space including all memories. Then with the increase of input strength, diffuse representation of higher category exhibits transitions to focused ones specific to each object. The hierarchy of memories is embedded in the transition probability from one memory to another during the spontaneous dynamics. With increased input strength, neural activity wanders over a narrower state space including a smaller set of memories, showing more specific category or memory corresponding to the applied input. Moreover, such coarse-to-fine transitions are also observed temporally during transient process under constant input, which agrees with experimental findings in the temporal cortex. These results suggest the hierarchy emerging through interaction with an external input underlies hierarchy during transient process, as well as in the spontaneous activity.

## Introduction

Categorization of objects in the environment according to their similarity is one of the fundamental functions of the human brain. It is typical to conceptualize categorization as involving a hierarchical structure: an object (e.g., A Doberman) belongs to a higher category (Dog), which, in turn, belongs to a much higher category (Animal). Unveiling how such hierarchical structure is represented in neural activity is essential to understanding the process of our memory and recognition. In spite of several studies that are related to the formation of categories with neural activities [1–4], however, neural dynamics underlying representation of hierarchical categories remain to be clarified.

In the traditional view, objects are represented as attractors in neural state space [5–7]. According to this view, the hierarchical structure of objects is embedded in the state space in such a way that each attractor corresponding to an object is located in a hierarchical way[5,8–14]. Attractors corresponding to objects in the same category are located nearby to form a cluster, which corresponds to the higher category. When these clusters of attractors belong to the same higher class of category, the clusters are again located nearby in the state space. The stability of attractors in such a hierarchical structure has been studied [9,14], and characteristic temporal behavior of neural activity also conforms with experimental observations [12,13].

Although the “representations-as-attractors” approach provides insight into categorization, the studies of this theoretical view have assumed that an external input is provided as the initial condition of neural activity and, consequently, determines a subsequent response, while external input itself does not reset neural activities as the initial condition in real neural system[15]. Recent experimental studies have revealed that intrinsic neural dynamics is highly organized even in the absence of external stimuli [16–18] and is markedly related to response of neural population to the external stimulus[19–22]. It is considered that responding neural activity results from interplay between the spontaneous dynamics and the external input. Under the “attractor” viewpoint, little attention has been paid to such interaction except few studies [23,24] and no studies focus on the hierarchy in the terms of interaction between spontaneous activity and external input. In this paper, we ask how hierarchical structure of categorized objects is represented in spontaneous activity, as well as the recall dynamics through the interaction.

To address this question, we modeled a recurrent neural network in which hierarchical memories are embedded based on “memories-as-bifurcations,” which is proposed in the previous studies[25,26]. This is a novel concept to understand possible relationship between spontaneous activity and representations of memories. Here, we focused on change in the flow structure in the neural state space with increasing the input strength. Our recurrent network model and the learning task are quite simple; combination of pairs of inputs and output targets are learned. With this simple setting, however, hierarchical clustering of neural activities depending on input strength is shaped by learning, and one can unveil basic logic underlying therein. We found that the neural dynamics representing a different level in the hierarchy of categories is produced as a function of input strength: Spontaneous activity spreads broadly over neural state space during which all memorized patterns emerge from time to time. Hierarchy of memories are embedded as the transition probability. It is interpreted that the spontaneous activity represents the highest category to which all memories belong, but not a specific one. With increasing input strength, the neural activity is focused around some memories in the same category. For further input strength, the neural activity converges to a target memory. Thus, successively descending level in the hierarchy is represented as successive bifurcations of the neural activity with increasing input strength.

This network also demonstrates the temporal transitions from the higher (coarse) to lower (fine) category representation, during transient process under an input for constant input strength, consistent with the recent experimental observation of temporal changes in neural activities from coarse-to-fine representations [2,4]. In the present paper, both the changes in the neural activity, one with an increase in the input strength and the other in time during transient process are shown to represent transitions from a higher to a lower category.

## Results

**Fig 1:**
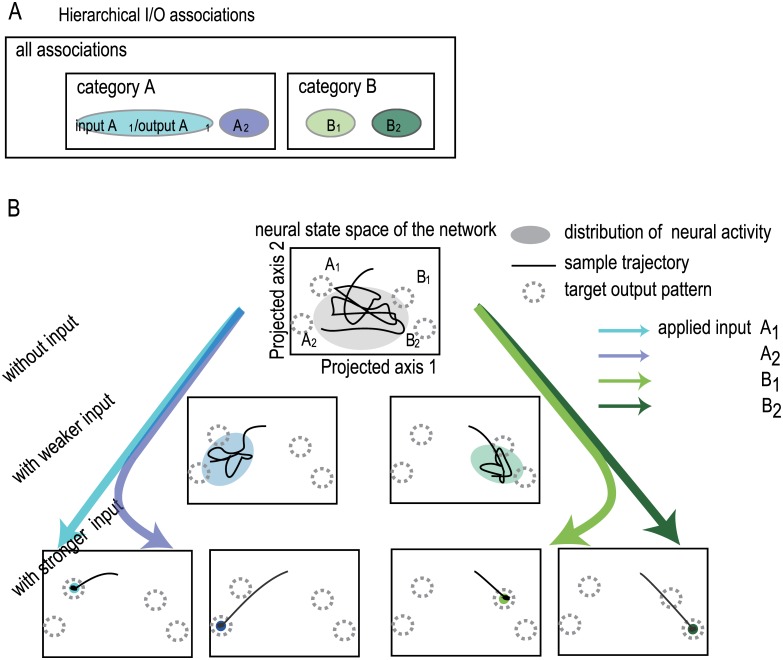
Schematic depiction of the dynamical systems view of categorization through bifurcation as a function of input intensity. A) Hierarchical structure of memorized input/ output target (I/O) associations. In the presence of an input, a network is trained for its activity pattern to represent the associated target. These associations are divided into categories A and B: associations $\eta_{}^{A_{1}}/\xi_{}^{A_{1}}$ and $\eta_{}^{A_{2}}/\xi_{}^{A_{2}}$ belong to the same category A, and their patterns are correlated, while associations A_1_ and B_1_ belong to different categories and their patterns are not correlated. See “Materials and Methods” for details. B) Representation of hierarchically structured associations with the change in input intensity. Under an input pattern $\eta_{}^{A_{1}}$ or $\eta_{}^{B_{1}}$ of a sufficient strength, neural activity is localized as an attractor at each of the associated target patterns (bottom panel). The neural activity represents each target pattern and thus this target is recalled successfully. With decreasing input intensity, the above attractor is no longer stable, and the neural activity diffuses to wander among the target patterns belonging to the same category (middle panel). The neural activities under the different input patterns in the same category are similar patterns, so that only 2 schematic figures for category A and B are plotted in the middle panel. The neural activity represents each of the categories A or B. With further decreasing input intensity, the neural activity outspreads over all the targets as spontaneous activity in the absence of inputs (top panel).

Before presenting the results from our model, we illustrate the dynamical systems’ view of hierarchical categorization to be presented here (see Fig 1). A network learns Input/Output (I/O) associations called *η*(*ξ*): each neuron (e.g., *i*-th neuron) in the network receives one input element (*η*_i_) and is required to generate *ξ*_i_. Note that an input pattern *η*_I_ and the corresponding target output pattern *ξ*_i_ are uncorrelated. Dynamics for neural activity *x*_*i*_ for neuron *i* are driven by interaction among neurons through synaptic connections, as well as an input pattern *η*_*i*_. We take a rate-coding model so that *x*_*i*_ is continuous. Thus, an input pattern does not perfectly determine the network state, but rather interplay between an input pattern and internal dynamics is critical to generate the adequate output pattern.

Synaptic matrix between neurons is changed so that the neural dynamics under an input generates an output that matches with the target pattern corresponding to the applied input (See “Materials and Methods” for the explicit neural and learning dynamics). Input patterns are randomly chosen but are correlated with each other in hierarchical manner, while target patterns are uncorrelated with input patterns, but are again correlated among the given hierarchical cluster. The learning process works so that for each input A_i_ (B_i_, C_i_,…) for the category A (B,C,,,), the corresponding target is generated as an output.

Now consider associations of A_1_ and A_2_ belong to the identical category A, and associations B_1_ and B_2_ belong to the category B (see subsection “Memory structure” below for details). After learning, the neural activity—upon presentation of a sufficiently strong input A_1_—is localized around the target pattern A_1_. The neural activity represents the target itself. With a weaker-intensity input A_1_, the neural activity changes in time, and approaches intermittently the targets A_1_ and A_2_, which belong to the category A. Hence, the evoked neural activity upon intermediate input A_1_ represents the category A including the target A_1_. With a further weakened input—or with no input—the neural activity is not focused on the corresponding category. The neural activity wanders across the target patterns (e.g., A_1_, A_2_, B_1_, B_2_). In this manner, depending on the input strength, the neural activity can code the target pattern, the category, and all the category’s items. We demonstrate numerically that the simple learning rule we have proposed produces this dynamic representation of the neural activity.

### Memory structure

We adopted a neural network of *N* (= 100) rate-coding neurons, whose synaptic matrix *J*_*ij*_ changes according to a learning rule (described in the subsection “Materials and Methods”) with which I/O associations are memorized in the network. Each of input and output patterns has as many elements as neurons (here, *N* = 100). In contrast to our previous study [25], we applied correlated patterns for inputs and targets forming the hierarchical structure, in which *M* associations are included in each of *K* categories, and a total of *M × K* associations is memorized. *M* input patterns as well as *M* target patterns are correlated within a category, while patterns across different categories are not correlated, and any pair of an input and a target pattern is not correlated. We established 6 categories (A–F), and each category contains 6 associations, giving a total of 36 I/O associations. Here, indices 0–5 associations belong to category A, and indices 6–11 belong to category B, and so forth.

### Recall of memory with strong input

**Fig 2:**
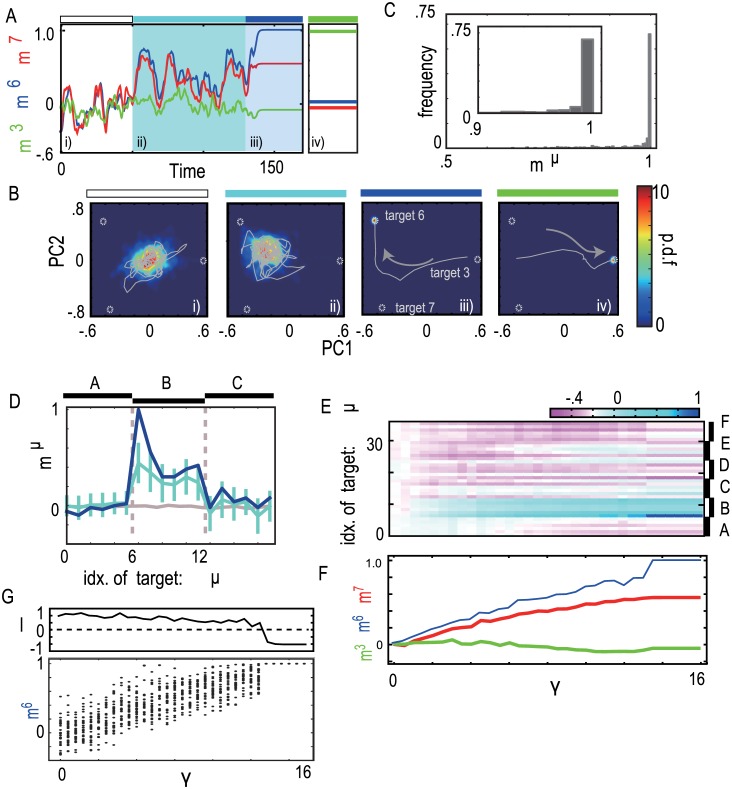
Change in the neural activity pattern against the increase of the input strength. A and B) The neural dynamics (i) without input, (ii) under the input 6 with strength *γ* = 6, (iii) with *γ* = 16, and (iv) under the input 3 with *γ* = 16, indicated by colored bars above figures in white, right blue, blue and green, respectively. A) The overlaps with the targets 6, 7, and 3 are plotted as blue, red, and green lines, respectively. Targets 6 and 7 are in same category B, while target 3 is in category A. B) The probability density distribution of the neural activity and a sample trajectory in principal component (PC) space. Dotted circles represent position of the targets. C) The distribution of the overlaps with the targets. We calculated the overlap with a target under the associated input with *γ* = 16 for 360 associations (36 associations in 10 networks). D) The temporal average overlap profiles for the spontaneous activity (gray) and for evoked activities with input strength *γ* = 6 (light blue) and 16 (dark blue). For calculating temporal average, we used overlaps over 400 unit time after 100 unit time transient. Error bars are standard deviation of neural activity over 400 unit time. In the following analysis, temporal average is calculated in this way unless otherwise mentioned. E,F) Change in overlap with increasing input is shown. The overlap with all of targets under input 6 against different strength is computed in E. As samples, the overlaps with targets 3,6, and 7 are plotted in F. G) Bifurcation diagram of the overlap with target 6 through increasing the input strength of input 6 (bottom) and the largest Lyapunov exponent (Top). We plot the overlaps at every 5 unit times over 250 unit times after transient time for each input strength.

We analyzed the neural dynamics after learning process in the following part. In order to represent neural activity dynamics, which has many variables, with a few relevant variables, we use the overlaps of the neural activity with input $m_{I}^{\mu }=\sum_{i}x_{i}\eta_{i}^{\mu }/N$ and target patterns $m_{T}^{\mu }=\sum_{i}x_{i}\xi_{i}^{\mu }/N$ (see the subsection “Materials and Methods”). In the following section, we mainly use $m_{T}^{\mu }$, denoting it as simply *m*^*μ*^. We first demonstrate that our learning process works well so that a given target is recalled upon the associated input with sufficient input strength *γ* (*γ* = *Γ* = 16, where *Γ* denotes the input strength used in the learning process. See “Materials and Methods”). As a typical example, the dynamics of evoked activities under inputs 3 and 6 are shown. By applying one of the learned input patterns, the overlap with the associated target surges to almost unity (Fig 2A(iii) and 2A(iv)) and an attractor that matches the target pattern emerges (Fig 2B(iii) and 2B(iv)). Thus, these targets are recalled successfully, consistent with our earlier study[25].

To examine the generality of these recall behaviors, we measured the overlap with the target *μ* under the associated input *μ* (μ = 0,⋯,35) in 10 networks (Fig 2C), each of which learns a different set of I/O associations. Almost all of overlaps (around 80%) take higher values over 0.9, meaning that almost all the targets were recalled successfully. Thus, the learning process works well and I/O associations are embedded successfully.

### Emergence of hierarchy with increasing input strength: From diffuse to focused representation

In the previous subsection, we showed that under a sufficiently strong input, a different attractor corresponding to the target pattern emerges depending on the applied input. We investigated how neural activity changed with increasing input strength in the following subsections. By using neural activity under input 6 as an example (Fig 2), we demonstrated that hierarchy of representation emerges: from representation of a category to that of a specific memory.

Fig 2A and 2B, show the neural activity dynamics during no input (i.e., spontaneous activity) and those under input pattern 6, with *γ* = 6 and with γ = 16. The spontaneous activity shows chaotic behavior (Fig 2A(i)) with a positive maximal Lyapunov exponent (Fig 2G) and shapes an attractor around the origin in the PC space (Fig 2B(i)). The neural activity approaches intermittently to the targets, which is analyzed later. Under the input with γ = 6, flow structure is changed (Fig 2B(ii)). A new attractor appears in the vicinity of both the target patterns 6 and 7. In fact, the overlaps of the evoked activity with targets 6 and 7 are elevated, while the overlap with target 3 remains low (Fig 2A(ii)). Here, targets 6 and 7 belong to the same category (called B), while 3 belongs to a different category (called A).

To more clearly show the relation between the evoked neural activity and targets depending on input strength, we computed a set of overlaps *m*^*μ*^ as a function of μ = 0,1,…,35 (= all memorized targets), which we refer to as the overlap profile (see “Materials and Methods”). Here, we calculated the temporal average overlap profile (Fig 2D). The profile line for the spontaneous activity is flat over all of targets. A network, thus, cannot distinguish targets each other, by overlap values. Then, the profile for *γ* = 6 shows that the overlaps with targets that belong to the same category as target 6 take distinctly higher values, as compared with other overlaps. The overlap profile is nearly flat against targets in category B, with the overlap values, 0.44±0.19, 0.35±0.19, 0.23±0.15, 0.22±0.17, 0.30±0.17, and 0.24±0.17 for target 6–11, respectively. Here, SD shows not the statistical error but the amplitude of temporal fluctuation around the mean value. Mean values plus one-SDs are comparable to the given correlation of targets (1, 0.56, 0.38, 0.3, 0.3, and 0.38, respectively), indicating that overlaps sometimes take values larger than the given correlation. Thus, the neural activity is sometime highly correlated with some of the targets in the category B beyond the given correlation of targets. Thus a network generates a neural activity which has a much higher similarity with targets in the category B than the expected by the given correlation structure. The produced output is interpreted not as representing only target 6, but representing the category B. Under a much stronger input value, the overlaps with targets 6 and 7 are separated (Fig 2A(iii)). The overlap profile shows that the neural activity matches well with target 6 and distinguishes it from other targets (Fig 2D).

To show the change in the neural activity by gradually increasing the input strength *γ*, we computed change of the overlaps with all of the targets (0, 1, …, 35) with increasing input strength (Fig 2E). For the intensity near zero, none of the overlap takes selectively a high value. As the intensity increases, the overlaps belonging to category B take higher values than those of the rest of overlaps in other categories. With increasing the strength further, the overlap with only target 6 is clearly higher than other overlaps. With increasing input strength, therefore, representation by the neural activity progresses from all targets to a category and then to a specific target. Finally, we confirmed generality of this change with increasing input strength in S1 File and S1 Fig.

### Hierarchical clusters of neural activity under different inputs

We, next, investigate how activities evoked by different inputs are organized with increasing input strength. Especially we will show that different clusters of these neural activities are generated in a hierarchical way depending on the input strength. For this purpose, we first demonstrate that neural activities for inputs 3,5,6 and 7 exhibit such hierarchical clustering, as an example and then analyze the entire behavior for all input patterns.

**Fig 3:**
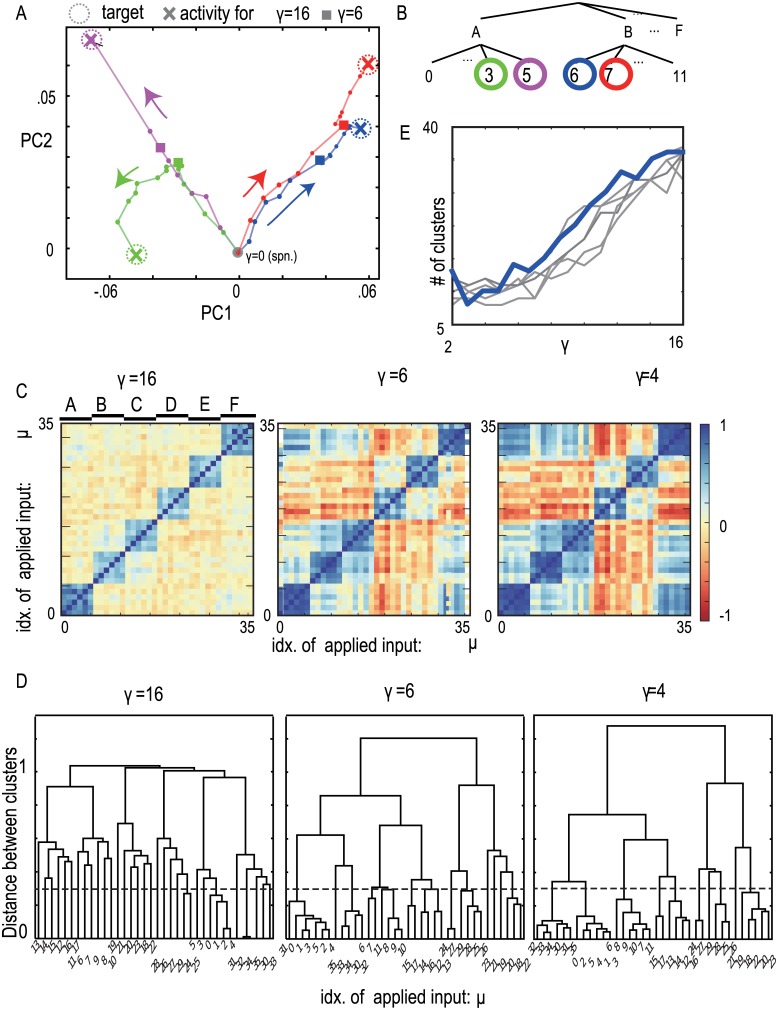
The neural activities under different input patterns with changing input intensity. A) Temporally average neural activity patterns projected in PC space. Spontaneous neural activity and evoked activities under input patterns 3 (green), 5 (magenta), 6 (blue), and 7 (red) with increase in input intensity are depicted. Bold points on lines show the neural activity patterns under the different input intensities from *γ* = 0 to *γ* = 16 by every 1 point. B) The hierarchy of embedded mappings is also plotted for reference, where the targets are marked in the same colors in the PC space. C) The similarity matrix *S*_*μν*_ between the evoked patterns under inputs *μ* and *ν* is plotted for *γ* = 4,6, and 16. Similarity is defined in the main text (see also detailed definition in”Materials and Methods”), whose (*μ*,*ν*) element indicates the similarity between the neural activities evoked by different inputs *μ* and *ν*. D) The cluster dendrogram of the similarity matrix in C. Dotted line shows threshold value (= 0.3) for counting the number of clusters. E) The number of the clusters as a function of the input strength. For each input strength, the number of clusters is obtained from the cluster dendrogram as in D. Bold line corresponds the number of the clusters for the network analyzed in above, and other lines show those for other networks.

Fig 3A shows temporal average activity patterns under input patterns 3, 5, 6, and 7 with increasing input intensity, represented in the PC space. Relationship between these associations are shown in a tree diagram in Fig 3B. For lower intensity of input, the neural activity patterns under inputs 6 and 7 (belonging to the same category B) are located closely with each other, while they are separated distinctly from those under inputs 3 and 5, which belong to a different category (i.e., A). These similar patterns are much more similar than expected by the given correlation structure as shown below. With increasing input strength, the activity patterns in the same category are separated. For γ = 16, the overlap under input 6 is distinct not only from those under inputs 3 and 5 but also from that under input 7. To quantitatively compare closeness, similarity between the neural activities under different inputs, μ and ν were computed by $S_{\mu v}=\sum_{i}^{}\bar{x_{i,\mu }}\bar{x_{i,v}}/\left|\bar{x_{\mu }}\right|\left|\bar{x_{v}}\right|$ where ${\bar{x}}_{i,\mu }$ denotes the temporal average activity of the *i-*th neuron under input μ, and $\left|\bar{x_{v}}\right|$ denotes the magnitude of $\bar{x_{v}}$. In this case, the similarities for γ = 6 are *S*_*36*_ = 0.065, *S*_*37*_ = 0.012, and *S*_*67*_ = 0.80. The similarities for γ = 16 are *S*_*36*_ = −0.02, *S*_*37*_ = 0.02, and *S*_*67*_ = 0.56. The similarities between targets themselves are *S*_*36*_ = −0.04, *S*_*37*_ = 0.04, and *S*_*67*_ = 0.56. Thus, for intermediate input strength, the neural patterns evoked by inputs 6 and 7 are more clustered than a given target cluster and hard to be distinguished, while for a higher strength, the neural patterns evoked by these inputs are more easily distinguished. In other words, the network responds similarly to different inputs in the same category when input is at an intermediate strength, while the network responds in a manner that allows the categories to be easily distinguished, even for inputs in the same category, when the input is stronger.

We also compared the neural activity under all the inputs memorized (*μ* = 0,1,⋯,35) to confirm the generality of the above behavior. Fig 3C diagrams similarity *S*_*μν*_ between the neural activity patterns evoked by inputs *μ* and *ν*, for input strengths 4, 6, and 16. Higher similarity *S*_*μν*_ indicates that a network generates similar neural patterns even under different inputs *μ* and *ν*. In these diagrams, a cluster formation that is dependent on the intensity of input is clearly visible. For *γ* = 16, each small cluster in the diagram corresponds to one of 6 categories A–F. This cluster structure exactly reflects that of the embedded targets (S2 Fig). As input strength is decreased to γ = 6, the degree of similarity in each category increases. Average similarity between neural patterns evoked by inputs in the same category is 0.71, while that between targets is ~0.49, which is about the value of imposed correlation. The average similarity between 0.49 and 1 indicates that similarity of neural patterns are determined not by solely correlation of the targets themselves, but is generated by the interplay between internal dynamics and an applied input. Decreasing the input strength to γ = 4 results in much higher similarity *Sμν* for inputs *μ* and *ν* in the same category (averaged similarity = 0.78), particularly for inputs A, B, and F. Interestingly, the similarity for neural activities not only between pairs in the same category, but also even between some pairs in different categories (A and F, B and C, and D and E) is selectively increased. This structure is not included in the given correlation structure of the target at all (S2 Fig). These clusters of categories are spontaneously generated with internal neural dynamics, through learning process. These clusters of categories can be considered as a higher level than the category level. Hence, a higher level in hierarchy is generated spontaneously as a result of memorizing simply correlated patterns.

Finally, we quantitatively characterized the similarity diagram by using the hierarchical cluster analysis (see“Materials and Methods”). We calculated the diagram from the above similarity matrix *S*_*μν*_ in Fig 3D and counted the number of clusters by setting a certain threshold for similarity distance (= 0.3 for all input strength). In Fig 3E, the number of clusters is plotted as a function of the input strength. For a weaker input strength, the number of clusters is around 10, implying that the neural activities under 36 different inputs are categorized into 10 groups. By increasing input strength, the number increases, until it reaches 31, nearly the number of learned associations, showing that each of the neural activities under different inputs is distinguishable from all others. To confirm if such behavior of the number of clusters is general, we computed the number of clusters for other ten networks and plotted in Fig 3E. The numbers of clusters in all networks are around 10 for a weaker input and with increasing input strength, the number increases more than 30.

In summary, neural activity patterns evoked by different inputs form a hierarchical structure. With increases in input strength, large clusters—within which neural activities evoked by different inputs are much more similar than the expected by correlation of targets—are successively separated into smaller clusters. Hence A network in our model represents a hierarchical category structure that depends on input strength. Note that this hierarchy is generally observed for another correlation parameter C as long as C is sufficiently larger (larger than around 0.16). See details in Supplemental information.

### Hierarchical structure embedded in spontaneous activity

Spontaneous neural activity in the absence of inputs shows chaotic oscillation (Fig 2A(i) and 2B(i)). As already discussed[16,19,20], spontaneous activity observed in neural system is not just noisy but also shows a structured spatio-temporal pattern in which specific patterns similar to stimulus-evoked patterns appear transiently[17,18]. It suggests that information about the stimulus-evoked patterns is potentially embedded in the spontaneous activity. In the present study, target patterns are embedded as evoked patterns in the presence of an input with sufficient strength. We then asked how a hierarchical category of these targets is embedded in the spontaneous neural activity, by comparing overlaps of spontaneous activity with target patterns.

**Fig 4:**
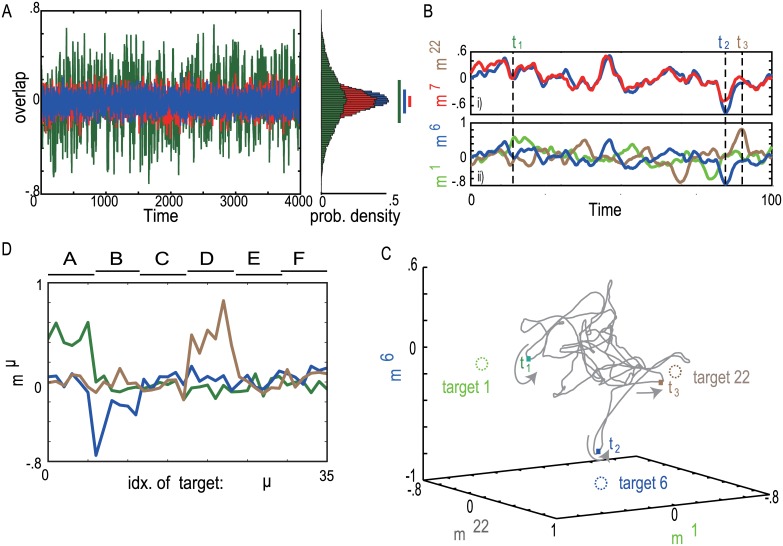
The neural dynamics in the spontaneous activity. A) Left: The overlaps of the spontaneous activity with target 1 (green) and with input 0 (blue) are plotted, while the overlap with a randomly generated binary pattern is plotted for reference in red. Right: The distribution of the overlap with targets, inputs and random patterns that are computed averaged over 36 patterns (μ = 0,1,**⋯**, 35) are shown. Color bars represent standard deviations of the distributions. B) The time series of the spontaneous neural activity is plotted. Time series of overlaps of the spontaneous activity with target 6 (blue) and 7 (red) are plotted in B(i), while that of the overlaps with target 6 (blue), 1 (green), and 22 (brown) are plotted in B(ii). C) The neural dynamics projected into three-dimensional space (each axis indicates the overlap with the target). Colored circles show the position of the target patterns. D) The overlap profile of the snapshot spontaneous activity at *t = t*_*1*_ (green) *t = t*_*2*_ (blue), and *t = t*_*3*_ (brown) for the time series shown in A.

We first examined whether spontaneous activity contains information of the target or input patterns. Here, (normalized) overlap of the neural activity with a given pattern provides a measure of distance between the generated neural activity and the pattern: If the overlap is closer to unity, the neural activity is closer to the pattern. In Fig 4A, the overlap of the spontaneous activity with a given target (target 1) intermittently takes a large value, while overlap neither with a non-memorized, randomly chosen pattern (green) nor with an input pattern (red) shows such intermittent increase and fluctuates around the value 0. This indicates that the spontaneous activity approaches the memorized target patterns more closely than other patterns.

To analyze closely relationship between the spontaneous activity and the target patterns, we again used an example, where the targets 6 and 7 belong to the same category B, the target 1 belongs to category A, and 22 belongs to category D. The overlaps of identical spontaneous activity with target patterns 6 and 7 (Fig 4B(i)) and those with target patterns 1, 6, and 22 (Fig 4B(ii)) are presented. The overlaps with target patterns 6 and 7 oscillate with high correlation with each other, although these show different peaks with slightly different timing. In contrast, the overlaps with target patterns 1, 6, and 22 do not show a correlated change. Approaches to these targets exhibit much larger different timing. The spontaneous activity approaches intermittently the different targets belonging to the different categories (Fig 4C) and we present the overlap profiles at these approaching times in Fig 4D. At *t = t*_*1*_, *t*_*2*_, and *t*_*3*_, the overlaps with the targets in the categories A, B, and D take high values in a target-selective fashion, respectively.

**Fig 5:**
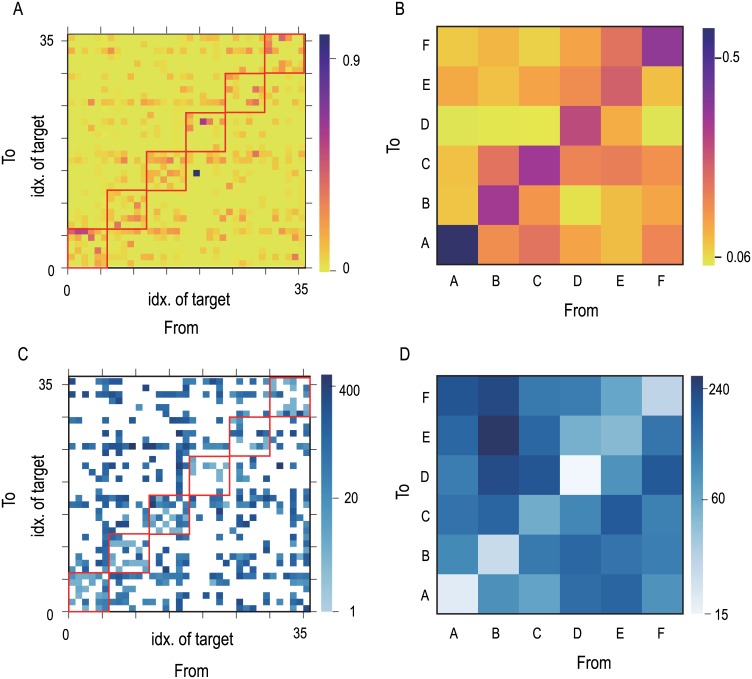
Temporal structure of spontaneous activity. A) Transition probability *P*_*μν*_ from the *ν*-th target to the *μ*-th target. We cannot compute the probability of self-visiting *P*_*μμ*_, and set at 0, because we did not distinguish continuous stay of the neural state around a target from coming in-out-in the identical target. B) Transition probability *P*_*ab*_ from the category b to the category a. C) Transition time *T*_*μν*_ from the *ν*-th to the *μ*-th target which is averaged with *P*_*μν*_. White tiles indicate that there is no transition and we cannot calculate the transition time. D) Transition time Tab from the target b to a. all of values are calculated from the spontaneous activity (0<t<10000). See “Materials and Methods” for the detailed.

Finally, we investigated how the spontaneous activity temporally approaches the targets within the same category and across different categories quantitatively. We calculated the transition probability *P*_*μν*_ (Fig 5A) and mean transition time *T*_*μν*_ from the *ν*-th to the *μ*-th target (Fig 5C) (See “Materials and Methods”). Spontaneous activity exhibited more frequent transitions often from one target to another (e.g., from target 2 to 5) than others that are less frequent (e.g., from target 2 to 35). To compare transition within a category and between categories, we calculated transition probability *P’*_*ab*_ and mean transition time matrix *T’*_*ab*_ among categories, as are plotted in Fig 5B and 5D, respectively. Spontaneous activity is highly likely to move within each of categories with shorter transition time, while it rarely jumps across categories and takes longer transition time. This means that the hierarchy of the targets is embedded in the temporal structure in the spontaneous activity.

### Hierarchical structure in transient dynamics: From diffuse to focused representation in time

So far, we have shown how neural representations change with increases in strength of an input in accordance with a hierarchical structure. We found that these transitions also occur temporally during the recall process under an input, when its input strength is constant. During recall, neural activity initially represents a category of several target patterns, and then converges to an attractor that represents a specific target.

**Fig 6:**
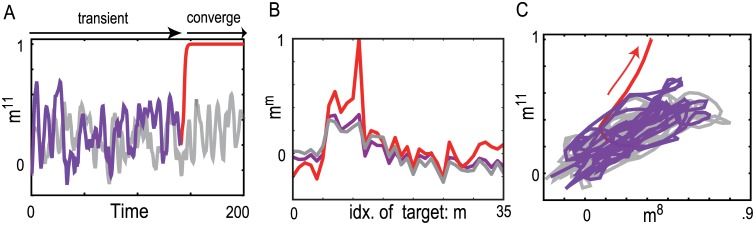
The transient neural activity before reaching the attractor. A-C) Transient dynamics of the neural activity under input 11 with *γ* = 5. The dynamics is colored before reaching the attractor in magenta and after convergence to the attractor in red. Neural activity after convergence to an attractor with a less intense strength (*γ* = 4) is plotted in gray, for reference. A) Time series of the overlap with the target pattern 11. B) Overlap profiles before and after convergence in magenta and red are plotted. Here, the former overlap profile is measured by averaging overlap after 50 unit-time transient up to the convergence point. The latter profile is measured after convergence. We also plot the profile for the neural activity with a less intense strength in gray as reference. C) The orbit of the neural activity dynamics in A is plotted, by projecting it into the two-dimensional space. The horizontal and vertical axes represent the overlaps with targets 8 and 11, respectively.

In Fig 6, we present recall dynamics until the neural activity converges on an attractor under input pattern 11 for γ = 5, which is slightly above the bifurcation point: under this point (γ = 4) an attractor in which the activity wanders among targets of a category is formed and, above this point γ = 5, a more localized pattern emerges as an attractor. The overlap of this neural activity with target 11 is plotted in Fig 6A. Up to *t* = 150, the neural activity fluctuates and then converges to a fixed-point attractor. In the transient fluctuation, the neural activity approaches not only target 11 but also other targets in the same category (Fig 6B). After this approach to the category, the neural activity converges on target 11 selectively. The neural activity pattern during the transient process reflects the attractor under a weaker input, where the neural activity is less localized and represents a coarser category. In fact, neural activity under input 11 after convergence to the attractor for γ = 4 (gray line in Fig 6) is highly overlapped with the transient neural activity when γ = 5 (magenta line in Fig 6). For many different initial conditions of trajectories, the neural activities show the same transition in time with different timing. Although, here, we demonstrate just one example, this temporal change is generally observed for different inputs (S3 Fig). In this way, the neural activity first approaches a state representing the category, before reaching the final attractor matching the single target, showing that the neural activity dynamics are organized hierarchically in time from a coarse to a specific representation.

## Discussion

It has recently been revealed that the interplay between spontaneous neural dynamics and external stimuli is significant in response behavior of neural activity[17–22]. We have proposed the novel viewpoint, “bifurcation memories through input strength” to understand this interplay[25,26]. By introducing a model that embodies this view, we focus on how hierarchical memories could be represented. We have shown that the neural dynamics generates their hierarchical structure from a coarse to a fine level with the increase in the input strength. Spontaneous activity in the absence of inputs shows successive approaches to and departures from each of the memories in the neural state space, representing the highest (coarsest) category to which all memories belong. By applying a learned input pattern with an intermediate strength, the evoked neural activity is localized near several targets, to represent the lower (finer) category to which a few associated memories belong. With further increases in the input strength, neural activity is localized only around a single target pattern associated with the applied input.

With increasing input strength, a network represents different level in hierarchy, (all targets -> a category -> a target). This hierarchy is included in a given correlation structure in I/O associations in the present model. However, by focusing carefully on the neural activities evoked by different inputs for smaller input strength shown in Fig 3, we found that the neural activities under different inputs in different categories, A and F, or B and C, exhibit higher similarity than correlation of these targets. This indicates that a network identifies these activities as a same pattern and a new level in hierarchy is generated through interplay between internal neural dynamics and inputs. Detailed study for the mechanism of spontaneous generation of new level is left as a future work.

Interestingly, we also found that the transition from coarse to fine categories is valid not only against the input strength but also in the temporal course of memory recall, which is consistent with previous experimental studies [2,4,27]. These studies have investigated how hierarchical objects are represented and processed in neural systems, especially in the visual system. In a visual system, many studies have revealed that the neural activity patterns showing coarse category emerge first and then fine category-related neural patterns arise in the process of identifying an object[2,27].

In our model, neural activity, in response to an input with a sufficient strength, first takes a broader distribution ranging over all the target patterns within the same category including the target to be recalled, before it converges on the target pattern. The broader distribution in the transient time represents the coarse category. Further, we have found that the temporally transient recall from a coarser to finer category reflects the bifurcation structure from the broader to localized attractor through the input strength. This result implies that the bifurcation structure in the intrinsic neural dynamics underlies the temporal processing from coarse to fine category observed in the experimental studies. Our proposition that relates the hierarchy to time and to input strength can be tested experimentally by observing neural activity dependence upon the input strength. This may be achieved by measuring the activity in the Inferior Temporal area [2] or olfactory bulb [28], as a function of input strength e.g., visual contrast or concentration of odors.

There are extensive studies on the conventional associative attractor memory with hierarchical structure, concerning dependence of the stability of memories upon their number [5,8,9,11,14] and also fluctuation on the weight matrix in terms of statistical physics [29]. Further, some studies [12,13] have proposed Hopfield-type models that embed hierarchical memories and exhibit the transient recall of mixed memories corresponding to coarse category. These, however, adopt a constant flow structure on neural state space with initial conditions determined by inputs. Hence relationship between neural activities with and without inputs cannot be discussed. Although some studies investigated this relationship [30–32], representation of hierarchical memories are not addressed. Rather, in the present study, we have revealed that a hierarchical structure of embedded memories in a network is reflected in the spontaneous activity, in bifurcation process with increasing the input strength and in the transient process during recall of a memorized pattern. These different representations of the structure of embedded memories have not been investigated in the previous studies. In addition, in our view, there is a single (or a few) attractor(s) for each of input strength and neural trajectories on neural state space from most of initial states converge to the required attractor, which is in contrast to the previous studies where trajectories from only neighborhood of the required attractor converge due to multiple attractors.

Finally, we discuss on the biological plausibility briefly. The learning rule we employ does not follow the Hebbian unsupervised fashion often used in standard recurrent neural-network models of the cerebral cortex [33], nevertheless it satisfies the minimum requirement for a biological learning rule in contrast to standard supervised learning fashion in recurrent networks [34,35]: According to one study [33], information available to a synapse is only local information for pre- and postsynaptic cells it directly connects and not any global information on cells it does not directly connect. In fact, our learning rule needs information only on the neural activity of the pre- and postsynaptic cells (*x*_*j*_ and *x*_*i*_, respectively) and the target signal to the postsynaptic cell *ξ*_*i*_. A possible situation might be considered to be a cortical circuit that receives bottom-up input from a lower sensory area or the thalamus and also top-down input from a higher area. This circuit is assumed to learn associations between these inputs and, after learning, reproduces the neural pattern matching with the top-down input pattern in response to the bottom-up input. Here, the top down input to an *i*-th neuron represents *ξ*_i_. This example is only one possible way to implement our model in a biological neural network, and future studies are needed to establish the model’s validity.

## Materials and Methods

### Patterns to be memorized

We let a network of *N*-neurons learn I/O associations, in which inputs called *η* and targets called *ξ* are binary patterns. In contrast to our previous study [25], we applied correlated patterns, which have a hierarchical structure, as follows: We generated *K* categories and *M × K* associations. We set 2*K* random patterns, *η*^*A*^ and *ξ*^*A*^, (A = 0,1,⋯,*K* − 1), as typical patterns of categories, where each element of a pattern takes a binary value generated with the probability *p*(*x*) = (1/2)*δ*(*x* − 1)+(1/2)*δ*(*x* + 1), and any pair of patterns is not correlated. Second, 2*M* members for each category are generated from the typical patterns, by flipping each element of the typical pattern with *p*_flp_. Thus, targets or inputs in the same category are correlated with *C* = (1 − 2*p*_flp_)^2^ on average and otherwise any pairs (including pairs of an input and its associated target) are not. We set *N* = 100, *K* = 6, *M* = 6, and *p*_flp_ = 0.15 so that *C* = 0.49. Within each category, inputs and targets are generated to satisfy $[\sum_{i}^{}\eta_{i}^{\nu }\eta_{i}^{\mu }/N]=[\sum_{i}^{}\xi_{i}^{\nu }\xi_{i}^{\mu }/N]=0.49$ for *μ*≠*v*, while otherwise, correlation between any patterns is chosen to be zero. Here, [⋯] represents the average over an ensemble of randomly generated patterns. Hence, 36 associations distributed among 6 categories are learned. Six categories *j* (*j* = 0,1, ···,5) is termed as A, B, C, ···, F in the order of *j*, and the associations μ = 6*j* + *l* (*l* = 0,1, ··,5) belong to the same category.

### Neural dynamics

We consider a system composed of *N* (= 100) continuous rate-coding neurons whose activity *x*_*i*_ (*i* = 1,2,···,*N*) lies between −1 and 1, basically identical to the network in our earlier study [25] and evolves according to

$$\dot{x}=F(\{x_{i}\},\{J_{ij}\})=\text{tanh}(\beta (\sum_{j\neq i}^{N}J_{ij}x_{j}+\gamma \eta_{i}^{\mu }))-x_{i}$$ (1)

where *J*_*ij*_ denotes a connection from the *j*-th to *i*-th neuron, *γη*^*μ*^ represents an input patternμwith strength *γ*, andμis the index of learned I/O associations. Parameter*β* is set at 4, respectively. We adopt the following learning procedure to embed the I/O associations.

### Learning procedure

The neural activity during learning process evolves in the presence of *γη* with a constant *γ* = Γ, while the connection *J*_*ij*_ is changed so that the neural activity pattern matches the target *ξ*. Note that *Γ* is used during learning process and *γ* is used in analysis of recall process after learning is completed. While *Γ* set at 16, we change *γ* between 0 and *Γ* to study the dependence of input strength on recall process. This evolution of the synaptic connection *J*_*ij*_ is given by

$$\dot{J}=\alpha (\xi_{i}-x_{i})x_{j}$$ (2)

where *α* > 0 is a learning parameter representing the rate of change in synaptic connections (relative to that of the neural activity). The above synaptic dynamics are determined by correlations between the activities of the pre- and postsynaptic neurons. This learning rule takes a similar form as the perceptron learning rule [36] where the synaptic connection is changed by correlations between activities of elements in the input and output layers. We set parameters at *α* = 0.01 and Γ = 16, since we have already confirmed that the network can learn a large number of memories with these parameter values (see [25]).

After an I/O association is learned according to (eq 2), the next association is learned. We train a network to memorize *MK* associations by applying this process iteratively *MK* × 100 times in a random order. During the learning process, both neural and synaptic dynamics run concurrently. The initial states are set as follows: the neural activities *x*_*i*_ take random values between −1 and 1 with a uniform probability, *J*_*ij*_ takes a random value from a binary ensemble of ±1 with equal probability. Fully connected networks without self-connections are used. For each run of different learning processes, different sets of mappings are learned so that the generated networks are different.

### Overlap analysis of neural dynamics

We define the following quantities to characterize the neural dynamics. The dynamics of the network are represented as a trail in *N*-dimensional neural state space; *N* is the number of neurons and is set at 100. *N*-dimensional trail is difficult to investigate directly, so we project these high-dimensional dynamics to a lower-dimensional state space by using the overlap of the neural dynamics with the inputs and targets: the overlaps with the input μ and the target μ are defined as $m_{I}^{\mu }=\sum_{i}x_{i}\eta_{i}^{\mu }/N$ and $m_{T}^{\mu }=\sum_{i}x_{i}\xi_{i}^{\mu }/N$, respectively. These values increase as the distance between the neural activity pattern and an input (or target) pattern is decreased, and they take unity when the distance vanishes. We use the overlap with a few of targets to understand how close the neural dynamics is to the concerned targets, denoted as *m*^*μ*^ (expressed, for simplicity, without the index *T*).

In contrast to single overlaps, we also use “overlap profile” to analyze global information. This is a function of overlap *m*^*μ*^ against *μ* = 0,1,⋯,(*KM* − 1) and represents how far the neural dynamics are to each of the targets. Let us first considering uncorrelated patterns. In this case, when the neural activity pattern is similar to one of targets, only the overlap with this target takes near-unity and others are close to zero, forming an overlap profile with a single peak. In contrast, when the neural dynamics do not approach any of the targets or approach all the targets equally, the overlaps with all the targets take near zero or a comparably high value, respectively, forming a flat overlap profile. In this way, the shape of the overlap profile indicates how much a given neural activity is localized to one (or a few) target pattern(s). This is the case for hierarchical patterns. Quantitative analysis is in Supporting information.

### Cluster analysis

In order to analyze how the neural activity is organized in response to different inputs of different input strength, we applied hierarchical cluster analysis against KM samples of neural activity under KM different input patterns for the same strength; here, KM = 36. In hierarchical cluster analysis, we chose two of the closest elements (a sample or a cluster of samples) and merged them into a larger cluster as a new element and this process was applied iteratively until the number of elements reached 1. To do this, we needed to define distance between elements (i.e., between a sample and a sample, a sample and a cluster, and a cluster and a cluster). In our analysis, samples were represented by neural activity patterns, and we defined the distance *D*_μν_ between the neural activity patterns under input *μ* and *ν* by defining the similarity *S*_μν_ as

$$S_{\mu \nu }=\sum_{i}{\bar{x}}_{i,\mu }{\bar{x}}_{i,\nu }/|{\bar{x}}_{\mu ,}{\bar{x}}_{\nu }|$$ (3)

$$D_{\mu \nu }=1-S_{\mu \nu }$$ (4)

We denoted the neural activity under input *ν* as *x*_*ν*_ and $\bar{x}$ represents the temporal average of *x*. We adopted the group average method as the criterion for forming the new larger cluster, using SciPy (see http://www.scipy.org/).

To study the effect of different input strengths, we examined the hierarchical analysis to plot dendrograms for *γ* = 4,6, and 16 in Fig 3D. We set the threshold at 0.3 under which the number of clusters is counted. The cluster number is computed for different input strengths and plotted for ten networks (Fig 3E). The value of the threshold is arbitrary, but the qualitative behavior of the cluster number against the input strength does not change.

### Transition probability in spontaneous activity

To analyze temporal structure of the spontaneous activity, we calculated which of the targets and when the spontaneous activity approaches. We first defined “approach to a target” as the neural state showing the overlap with this target larger than 0.5. (If more than one targets give overlaps of more than 0.5 simultaneously, a target with the highest overlap is defined as the approached one). By this definition, we computed set of time {*t*_*i*_} (0< *t*_*0*_<*t*_*1*_<…) at which the spontaneous activity approaches a target most closely. A approached target at *t*_*i*_ is denoted as *α*_*i*_ (*i* = 0,1,,,35). We calculated sequences of the approach time {*t*_*i*_} and of approached target {*α*_*i*_} from spontaneous activity (0<t<10000). From these sequences, we calculated the transition probability *P*_*μν*_ (*Σ*_*μ*_*P*_*μv*_ = 1) and mean transition time *T*_*μν*_ from the *ν*-th to the *μ*-th target. Further, we computed transition probability *P’*_*ab*_ and mean transition time *T’*_*ab*_ from category b to a according to

$$P_{ab}=(\sum_{\mu \in a,\nu \in b}\delta \left(\alpha_{i+1}=\mu \right)\delta \left(\alpha_{i}=\nu \right))/\sum_{\mu ,\nu \in b}\delta \left(\alpha_{i+1}=\mu \right)\delta \left(\alpha_{i}=\nu \right),$$ (5)

$$T_{ab}=(\sum_{\mu \in a,\nu \in b}\left(t_{i+1}-t_{i}\right)\delta \left(\alpha_{i+1}=\mu \right)\delta \left(\alpha_{i}=\nu \right))/\sum_{\mu ,\nu \in b}\delta \left(\alpha_{i+1}=\mu \right)\delta \left(\alpha_{i}=\nu \right)$$ (6)

## Supporting Information

S1 Fig Neural dynamics with increasing input strength. A) Schematic image of the localization factor σ. The factor indicates how the overlap profile becomes concentrated on fewer targets and then how the neural activity is localized around these targets. B) The upper figure is same as Fig 2E for comparing with the localization factor. In lower figure, the normalized localization factor averaged over 200 unit-time-steps is plotted. Each line indicates change in the localization factor for neural activity response to different input patterns. Blue line corresponds to the factor computed from Fig A.
(EPS)

S2 Fig Similarity between different patterns. A) Similarity matrix between different targets that are prescribed (Left) and inputs that are applied(Right). Similarity matrix is measured in the same way as in Fig 3C. B) Deviation of similarity between evoked neural patterns against inputs from the similarity in the target patterns. Each element in the matrix is defined as *S*_*μv*,activity_ − *S*_*μv*,target_. Here, *S*_*μv*,activity_ and *S*_*μv*,target_ are similarity between evoked activities under input μ and ν and similarity between targets μ and ν, respectively. See details in “Materials and Methods.”
(EPS)

S3 Fig Transient dynamics under different inputs. A(i) Transient dynamics of the neural activity under input 11 as same as Fig 6A for reference. (ii) Time series of the temporally average localization factor. The temporal average of the localization factor was computed with a sliding window of 20 unit-time-steps. B) overlap with target 7 for input strength γ = 11 and its localization factor as same as in A. C) The time series of the localization factor for neural activity under different input patterns. The black bold line corresponds to the example used in A and B, while gray lines show the time series of the factor upon the inputs other than 7 and 11.
(EPS)

S4 Fig Similarity matrix for different parameters. AB) The similarity matrix *S*_*μν*_ between the evoked patterns for different correlation parameters, corresponding to Fig 3C. Similarity matrix for the correlation parameter 0.36. B) That for the correlation parameters = 0.16. C) Deviation of the similarity of the evoked patterns under inputs in the same category from correlation of targets for different correlation parameters. The deviation is calculated as Σ_*μ*,*v* in same category_(*S*_*μv*,activity_ − *S*_*μv*,target_)/(*M*(*M* − 1) × *K*). *S*_*μv*,activity_ and *S*_*μv*,target_ are same as in S2 Fig. *M* and *K* are the number of members in each category and that of categories, respectively.
(EPS)

S1 File Supporting analysis. Generality of dynamic hierarchy against the change in input strength.Comparison between dynamics structure and a given memory structure.Generality of temporal hierarchy.Neural behavior for different parameters.Supplemental methods.
(DOCX)

## References

[1] Freedman DJ, Assad J a. Experience-dependent representation of visual categories in parietal cortex. Nature. 2006;443: 85–8. 10.1038/nature05078 16936716

[2] Sugase Y, Yamane S, Ueno S, Kawano K. Global and fine information coded by single neurons in the temporal visual cortex. Nature. 1999;400: 869–73. 10.1038/23703 10476965

[3] Ohl F, Scheich H, Freeman WJ. Change in pattern of ongoing cortical activity with auditory category learning. Nature. 2001;412: 733–6. 10.1038/35089076 11507640

[4] Hegdé J. Time course of visual perception: coarse-to-fine processing and beyond. Prog Neurobiol. 2008;84: 405–439. 10.1016/j.pneurobio.2007.09.001 17976895

[5] Amari S. Neural Theory of Association and Concept-Formation. Biol Cybern. 1977;185: 175–185. 10.1007/BF00365229 901864

[6] Hopfield JJ. Neurons with graded response have collective computational properties like those of two-state neurons. Proc Natl Acad Sci U S A. 1984;81: 3088–92. 6587342 10.1073/pnas.81.10.3088 PMC345226

[7] Amit DJ. Modeling Brain Function: The World of Attractor Neural Networks. Cambridge University Press; 1992.

[8] Cortes C, Krogh AS, Hertz J. Hierarchical associative networks. J Phys A Math Gen. 1987;20: 4449–4455.

[9] Bacci S, Mato G, Parga N. Dynamics of a neural network with hierarchically stored patterns. J Phys A Math Gen. 1990;23: 1801–1810. 10.1088/0305-4470/23/10/020

[10] Engel A. Enlarged basin of attraction in neural networks with persistent stimuli. Phys Rev A. 1990;42. 10.1103/physreva.42.4998 9904612

[11] Cartling B. Dynamics control of semantic processes in a hierarchical associative memory. Biol Cybern. 1996;71: 63–71. 10.1007/BF00199138 8573654

[12] Toya K, Fukushima K, Kabashima Y. Bistability of mixed states in a neural network storing hierarchical patterns. J Phys A Math Gen. 2000;33: 2725–2737.

[13] Matsumoto N, Okada M, Sugase-Miyamoto Y, Yamane S. Neuronal mechanisms encoding global-to-fine information in inferior-temporal cortex. J Comput Neurosci. 2005;18: 85–103. 10.1007/s10827-005-5476-4 15789171

[14] Gutfreund H. Neural networks with hierarchically correlated patterns. Phys Rev A. 1988;37: 570–577. 10.1103/physreva.37.570 9899687

[15] Fiser J, Chiu C, Weliky M. Small modulation of ongoing cortical dynamics by sensory input during natural vision. Nature. 2004;431: 573–578. 15457262 10.1038/nature02907

[16] Mao B-Q, Hamzei-Sichani F, Aronov D, Froemke RC, Yuste R. Dynamics of Spontaneous Activity in Neocortical Slices. Neuron. 2001;32: 883–898. 10.1016/S0896-6273(01)00518-9 11738033

[17] Kenet T, Bibitchkov D, Tsodyks M, Grinvald A, Arieli A. Spontaneously emerging cortical representations of visual attributes. Nature. 2003;425: 954–956. 14586468 10.1038/nature02078

[18] Luczak A, Bartho P, Harris KD. Spontaneous Events Outline the Realm of Possible Sensory Responses in Neocortical Populations. Neuron. 2009;62: 413–425. 10.1016/j.neuron.2009.03.014 19447096 PMC2696272

[19] Ringach DL. Spontaneous and driven cortical activity: implications for computation. Curr Opin Neurobiol. 2009;19: 439–444. 10.1016/j.conb.2009.07.005 19647992 PMC3319344

[20] Berkes P, Orbán G, Lengyel M, Fiser J. Spontaneous cortical activity reveals hallmarks of an optimal internal model of the environment. Science. 2011;331: 83–7. 10.1126/science.1195870 21212356 PMC3065813

[21] MacLean JN, Watson BO, Aaron GB, Yuste R. Internal Dynamics Determine the Cortical Response to Thalamic Stimulation. Neuron. 2005;48: 811–823. 16337918 10.1016/j.neuron.2005.09.035

[22] Luczak A, Bartho P, Harris KD. Gating of Sensory Input by Spontaneous Cortical Activity. J Neurosci. 2013;33: 1684–1695. 10.1523/JNEUROSCI.2928-12.2013 23345241 PMC3672963

[23] Siri B, Berry H, Cessac B, Delord B, Quoy M. A mathematical analysis of the effects of Hebbian learning rules on the dynamics and structure of discrete-time random recurrent neural networks. Neural Comput. 2008;20: 2937–66. 10.1162/neco.2008.05-07-530 18624656

[24] Deco G, Hugues E. Neural network mechanisms underlying stimulus driven variability reduction. PLoS Comput Biol. 2012;8: e1002395 10.1371/journal.pcbi.1002395 22479168 PMC3315452

[25] Kurikawa T, Kaneko K. Embedding responses in spontaneous neural activity shaped through sequential learning. PLoS Comput Biol. 2013;9: e1002943 10.1371/journal.pcbi.1002943 23505355 PMC3591288

[26] Kurikawa T, Kaneko K. Associative memory model with spontaneous neural activity. EPL (Europhysics Lett. 2012;98: 48002.

[27] Hegdé J, Van Essen DC. Temporal dynamics of shape analysis in macaque visual area V2. J Neurophysiol. 2004;92: 3030–42. 10.1152/jn.00822.2003 15201315

[28] Niessing J, Friedrich RW. Olfactory pattern classification by discrete neuronal network states. Nature. 2010;465: 47–52. 10.1038/nature08961 20393466

[29] Virasoro M. The Effect of Synapses Destruction on Categorization by Neural Networks. EPL (Europhysics Lett. 1988;7: 293–298.

[30] Abbott LF, Rajan K, Sompolinsky H. Interactions between Intrinsic and Stimulus-Evoked Activity in Recurrent Neural Networks. 2009; 1–16.

[31] Goldberg JA, Rokni U, Sompolinsky H. Patterns of Ongoing Activity and the Functional Architecture of the Primary Visual Cortex. Neuron. 2004;42: 489–500. 10.1016/S0896-6273(04)00197-7 15134644

[32] Marre O, Yger P, Davison AP, Fregnac Y. Reliable Recall of Spontaneous Activity Patterns in Cortical Networks. J Neurosci. 2009;29: 14596–14606. 10.1523/JNEUROSCI.0753-09.2009 19923292 PMC6665826

[33] Fusi S. Hebbian spike-driven synaptic plasticity for learning patterns of mean firing rates. Biol Cybern. 2002;87: 459–470. 12461635 10.1007/s00422-002-0356-8

[34] Sussillo D, Abbott LF. Generating coherent patterns of activity from chaotic neural networks. Neuron. Elsevier Ltd; 2009;63: 544–57. 10.1016/j.neuron.2009.07.018 PMC2756108 19709635

[35] Williams RJ, Zipser D. A Learning Algorithm for Continually Running Fully Recurrent Neural Networks. Neural Comput. MIT Press; 1989;1: 270–280. 10.1162/neco.1989.1.2.270

[36] Dayan P, Abbott LF. Theoretical Neuroscience: Computational and Mathematical Modeling of Neural Systems. Cambridge, MA: MIT Press; 2001.

