# Supplementary material for: Dynamic Organization of Hierarchical Memories
Source: PLoS One. 2016 Sep 12;11(9):e0162640. doi: 10.1371/journal.pone.0162640 (PMC5019405; doi:10.1371/journal.pone.0162640)
Supplement: S1 File — Generality of dynamic hierarchy against the change in input strength.Comparison between dynamics structure and a given memory structure.Generality of temporal hierarchy.Neural behavior for different parameters.Supplemental methods. (DOCX) [file pone.0162640.s005.docx]

Supporting analysis

Title: **Dynamic organization of hierarchical memories**

Abbreviated title: **Dynamic organization of hierarchical memories**

Authors: Tomoki Kurikawa^1,3,4^ and Kunihiko Kaneko^2^

1. **Generality of dynamic hierarchy against the change in input strength.**

As given in Fig. 2, the evoked activity under weak input exhibits a diffuse neural pattern across different targets in the same category under weak input, while, under stronger input, the activity shows a focused pattern representing a target. We examine generality of this change depending on input strength by introducing a localization factor. The localization factor gives a measure of how much neural activity is focused around one (or a few) target patterns (See “Supplemental methods” in detail). Consider general uncorrelated patterns as an example. When the neural activity matches one of the targets (green line in S1A Fig), the factor is close to unity. A low value of factor σ indicates less precise matching: when the activity pattern is equally far from all target patterns (black line in S1A Fig), the factor becomes the inverse of the number of targets. In the following analysis, we use the normalized factor σ/σ_target_.

We computed the normalized factor by increasing input strength (S1B Fig). The factor increases gradually and then discontinuously elevates at the bifurcation point, confirming that the neural activity is localized only to the target by the increase in input strength. To examine whether the observed categorization process is general, we measured the factor under a different input pattern against the input strength γ (S1B Fig). In almost all values of γ, the indices gradually increase with the input strength γ, and, then, at a certain value, they show a rapid increase toward unity. Generally, the neural activity for medium input strength approaches several targets belonging to the same category, while for the stronger input, it approaches selectively to the target to be recalled.

1. **Comparison between dynamics structure and a given memory structure.**

We found that neural activities under different inputs in the same category are quite similar for smaller input strength and claimed that neural dynamics can represent different level in hierarchy, i.e., category and target pattern level, depending on the input strength. In our model, however, targets themselves are hierarchically correlated and there is the possibility that the similarity of neural activities is just reflected from the static correlation of targets. To check the difference between similarities of neural activities and targets, we compare the similarity between the targets and the neural activities for different input strength in S2 Fig. For much higher strength (*γ*=16), the similarity matrix of the neural activities almost matches that of the targets, because a network retrieves most of memories. For lower input strength, in contrast, the large difference is observed in the same category. In addition, the similarity between the neural activities belonging to different categories (A,B, C and F), or (D and E), exhibits much higher value than that of the targets. These indicates that, for lower input strength, the similarity of neural activity is not determined only be the target structure, but also is formed through interplay between the internal dynamics and the applied input.

1. **Generality of temporal hierarchy.**

We showed in Fig. 6 that the neural activity first approaches a state representing the category, before reaching the final attractor matching the single target. In this way, the neural dynamics are organized hierarchically temporally from a coarse to a specific representation. Although, in Fig. 6, we demonstrated only one example, we, here, examine generality of this temporally organized hierarchy. For this purpose, we investigated the neural activity for different inputs. To quantify the transition from diffuse to focused pattern, we computed temporal averaged localization factor $\bar{\sigma}/\sigma_{target}$ with moving window of averaging as the above. We exhibit two examples of neural activities and localization factor in S3A and B Figs. The averaged localization factor changes from low value representing diffuse pattern to higher value corresponding to focused pattern at transition point of the overlap behavior. The time course of the localization factor during the transient process is plotted for different input patterns with strengths that are sufficient to generate recall of a single target (specified values of strength are dependent on the applied inputs). The rapid change from low to high value, representing transition from diffuse to focused pattern, is observed for trajectories.

1. **Neural behavior for different parameters.**

We investigated neural behavior for the following parameters which are different from that in the main text in order to confirm generality of the neural behavior we found. Parameters values are same as those in the main text, unless otherwise mentioned.

a) Learning rate *α* and input strength *Γ* (this *Γ* denotes input strength used in the learning process which is different from *γ* used in recall process): in the previous study, we have analyzed dependence of learning rate and input strength on neural behavior. We have found that there are two regimes, single memory regime and multiple memory regime. In the current study, we have chosen (*α,Γ*)=(0.01, 16) in the multiple memory regime, so that multiple targets are memorized. We investigated memory capacity (here, the number of targets, overlap with which is larger than 0.9) in the multiple memory regime in Table 1, because in the present study memorized I/O associations are correlated hierarchically and iteratively learned, so that the capacity is different from the case to memorize uncorrelated patterns as studied previously. These results indicate that a network can learn multiple memories well for these parameters.

b) Correlation parameter C: first, we computed memory capacity for different correlation parameters in Table 1b. For all parameters, the capacity is quite high.

Next, we analyzed whether the neural activity exhibits hierarchy in clusters depending on input strength. Similarity matrix among neural activity patterns for different applied input for γ=4 is shown in S4A Figs, where the correlation value C is set at 0.36. As shown, different inputs in the identical category evoke similar neural patterns and thus hierarchical category structure is shaped in the same way as in the case with C=0.49 shown in Fig. 3C. The similarity is generated by interplay between the internal dynamics and inputs, not only by the correlation of targets. Actually, the similarity in the same category is larger than correlation of targets. For large input strength (γ=16), similarity between neural activities are determined by correlation of targets. Thus, dynamic organization in hierarchy is shaped in the same way as for C=0.49 in the main text.

For C=0.16, in contrast, the similarity in the same category is hardly shaped for *γ*=4 as shown in B. To examine how correlated activities are shaped against the inputs in the same category, we computed how much the similarity between evoked neural patterns against inputs in the same category is deviated from the similarity in the target patterns. This deviation of the similarity from target’s correlation is 0.32 for C=0.36, and 0.15 for C=0.16. We plot the deviation for different C in S4C Fig. Below C=0.25, deviation is drastically decreased, indicating that above 0.25, the similarity in the same category is much larger than the correlation of targets. These results show that when applied correlation is larger, the evoked neural activity shapes categories across similar input patterns, as represented in the neural dynamics.

**5. Supplemental methods**

Definition of the localization factor:

To quantify how fragile the overlap profile is, we introduced a localization factor σ defined as:

$\sigma=\sum_{\mu}^{MK-1} \left( m^{\mu} \right)^{4}/(\sum_{\mu}^{MK-1} {(m^{\mu})}^{2})^{2}$.

In general, if an overlap profile is concentrated on one element (m^ν^= 1 and m^μ^ = 0 for μ≠ν), the factor takes unity. When it is extended to all elements (m^μ^= 1 for all), then the factor is the inverse of the number of elements. Furthermore, this value is not changed when all m^μ^ are multiplied uniformly by some constant. In a case of correlated targets, when the neural activity pattern is similar to a single target pattern, the factor does not take unity because all of other elements are not zero due to correlation among targets. In order to normalize the factor, we computed a normalized factor σ/σ_target_ that is computed by dividing the primitive factor σ by that for the target pattern σ_target_ to be recalled.

Table 1a: Memory capacity

| input strength  learning rate | 10 | 16 |
| --- | --- | --- |
| 0.01 | 0.69 | 0.79 |
| 0.005 | 0.86 | 0.87 |

Table 1b:

| C=0.36 | C=0.25 | C=0.16 |
| --- | --- | --- |
| 0.89 | 0.92 | 0.94 |

S1 Fig: Neural dynamics with increasing input strength.

A) Schematic image of the localization factor σ. The factor indicates how the overlap profile becomes concentrated on fewer targets and then how the neural activity is localized around these targets. B) The upper figure is same as Fig2 E for comparing with the localization factor. In lower figure, the normalized localization factor averaged over 200 unit-time-steps is plotted. Each line indicates change in the localization factor for neural activity response to different input patterns. Blue line corresponds to the factor computed from Fig A.

S2 Fig: Similarity between different patterns. A) Similarity matrix between different targets that are prescribed (Left) and inputs that are applied(Right). Similarity matrix is measured in the same way as in Fig. 3C. B) Deviation of similarity between evoked neural patterns against inputs from the similarity in the target patterns. Each element in the matrix is defined as $S_{\mu\nu,\text{activity}}-S_{\mu\nu\text{,target}}$. Here, $S_{\mu\nu,\text{activity}}$ and $S_{\mu\nu\text{,target}}$ are similarity between evoked activities under input μ and ν and similarity between targets μ and ν, respectively. See details in “Materials and Methods.”

S3 Fig: Transient dynamics under different inputs.

A(i) Transient dynamics of the neural activity under input 11 as same as Fig 6A for reference. (ii) Time series of the temporally average localization factor. The temporal average of the localization factor was computed with a sliding window of 20 unit-time-steps. B) overlap with target 7 for input strength γ=11 and its localization factor as same as in A. C) The time series of the localization factor for neural activity under different input patterns. The black bold line corresponds to the example used in A and B, while gray lines show the time series of the factor upon the inputs other than 7 and 11.

S4 Fig: Similarity matrix for different parameters. AB) The similarity matrix *S_μν_* between the evoked patterns for different correlation parameters, corresponding to Fig. 3C. Similarity matrix for the correlation parameter 0.36. B) That for the correlation parameters = 0.16. C) Deviation of the similarity of the evoked patterns under inputs in the same category from correlation of targets for different correlation parameters. The deviation is calculated as $\text{ }\sum_{\mu,\nu\text{ in same category}} {(S}_{\mu\nu, \text{activity}}-S_{\mu\nu,\text{target}})/(M(M-1)\times K)$. $S_{\mu\nu,\text{activity}}$ and $S_{\mu\nu,\text{target}}$ are same as in S2 Fig. *M* and *K* are the number of members in each category and that of categories, respectively.
